# Parasites: The Inside Story

**DOI:** 10.3201/eid2910.230235

**Published:** 2023-10

**Authors:** Rezhan H. Hussein

**Affiliations:** Penn State College of Medicine, Hershey, Pennsylvania, USA

**Keywords:** parasites, climate change, changing ecosystems, vector-borne infections, viruses, zoonoses, book review

Parasites: The Inside Story shows how parasites coexisted and spread around the world through host migration, particularly through humans, and researches their origin and evolution (Figure). The book describes how the choice of host has affected the successful survival of some parasites and avoidance of extinction. The authors show how parasites influence and manipulate their intermediate host, which makes them more visible and easier prey for the next host, causing the host to “look foolish.” The authors use novel and entertaining approaches to explore their subject, such as describing how the Israelite Jonah spent 3 days in the belly of a whale and what parasites he might have encountered during that 3-day stay. 

**Figure:**
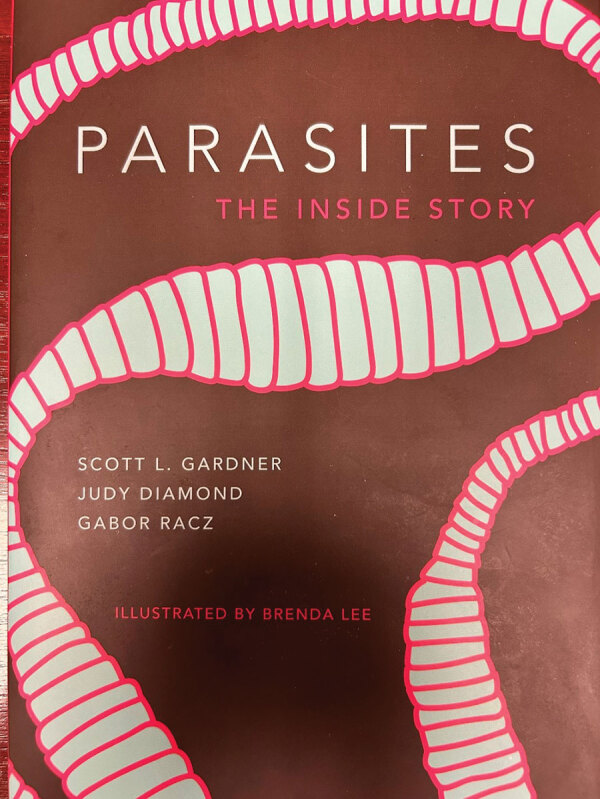
Parasites: The Inside Story

Another entertaining approach was participation in the parasite Olympics, where parasites earned medals according to their success and survival by choosing and adjusting to their hosts. *Ascaris lumbricoides* is a gold medal winner as one of the most durable internal parasites of humans. However, the book overlooked *Strongyloides stercoralis*, which can cause hyperinfection syndrome in immunocompromised hosts and is considered an emerging infectious disease and deserves an honorary Olympic medal. Babesiosis is another notable emerging parasitic disease that was overlooked.

I appreciated the novel and appealing way in which the authors show how parasites affect other species and human lives, while also incorporating their nonharmful roles, as in the case of mutualism and commensalism. I loved the immense and vivid imagery used to describe the breathtaking basin of the Congo River but was saddened that many persons living along the river might not continue to see that beautiful scenery because of river blindness caused by *Onchocerca volvulus*. The stories, connections to real persons, and references to at least 3 movies and 1 television series indicate the detailed efforts involved in writing this book.

The book has a high level of scientific detail as it dives into history, ecology, evolution, and future outlooks. The authors discuss how climate change is affecting parasite existence. An example is the migration of triatomine kissing bugs to the southern half of the United States, which might increase the risk for *Trypanosoma cruzi* transmission, causing Chagas disease (to which the book attributes Charles Darwin’s death, from an infection he acquired during a trip to South America). The authors also elaborate on international scientific collaboration during epidemiologic investigations, such as the collaboration between researchers from the United States and Mongolia during a hantavirus infection outbreak in New Mexico. Readers will learn about parasite eradication campaigns, some of which did not turn out well, as was the case for the *Schistosoma mansoni* eradication campaign in Egypt that resulted in populationwide infections with hepatitis C virus.

The book has 29 color photos, making the stories only more vibrant, and helps in understanding parasite life cycles and in making diagnoses. This book will appeal to readers interested in emerging infectious diseases, nonparasitologists, scientists, and clinicians and is an admirable expedition into the amazing world of parasites.

